# Identification of key amino acid residues responsible for internal and external pH sensitivity of Orai1/STIM1 channels

**DOI:** 10.1038/srep16747

**Published:** 2015-11-18

**Authors:** Hiroto Tsujikawa, Albert S Yu, Jia Xie, Zhichao Yue, Wenzhong Yang, Yanlin He, Lixia Yue

**Affiliations:** 1 Calhoun Cardiology Center, Department of Cell Biology, University of Connecticut Health Center, Farmington, CT, USA

## Abstract

Changes of intracellular and extracellular pH are involved in a variety of physiological and pathological processes, in which regulation of the Ca^2+^ release activated Ca^2+^ channel (I_CRAC_) by pH has been implicated. Ca^2+^ entry mediated by I_CRAC_ has been shown to be regulated by acidic or alkaline pH. Whereas several amino acid residues have been shown to contribute to extracellular pH (pH_o_) sensitivity, the molecular mechanism for intracellular pH (pH_i_) sensitivity of Orai1/STIM1 is not fully understood. By investigating a series of mutations, we find that the previously identified residue E106 is responsible for pH_o_ sensitivity when Ca^2+^ is the charge carrier. Unexpectedly, we identify that the residue E190 is responsible for pH_o_ sensitivity when Na^+^ is the charge carrier. Furthermore, the intracellular mutant H155F markedly diminishes the response to acidic and alkaline pH_i_, suggesting that H155 is responsible for pH_i_ sensitivity of Orai1/STIM1. Our results indicate that, whereas H155 is the intracellular pH sensor of Orai1/STIM1, the molecular mechanism of external pH sensitivity varies depending on the permeant cations. As changes of pH are involved in various physiological/pathological functions, Orai/STIM channels may be an important mediator for various physiological and pathological processes associated with acidosis and alkalinization.

A variety of physiological and pathological processes are regulated by alterations in intracellular and extracellular pH^1^,^2^. For example, intracellular alkalinization is associated with physiological functions such as activity-dependent membrane depolarization^3^, oocyte maturation^4^, sperm activation^5^,^6^,^7^, and growth factor induced cell proliferation, differentiation, migration, and chemotaxis^1^. Pathologically, intracellular alkalinization and extracellular acidosis are hallmarks of malignant cells and are associated with tumor progression^8^,^9^, and intracellular acidic pH (pH_i_) has been shown to promote apoptosis^10^. Extracellular low pH, which occurs under injury and ischemia conditions, inhibits a number of cellular responses, including cytosolic- and membrane-associated enzyme activities, and ion transport as well as ion channel activities^2^. Many cases of clinical acidosis are also accompanied by immunodeficiency^2^.

Considerable evidence has accumulated that Ca^2+^ signaling is involved in various physiological/pathological processes associated with acidosis and alkalinization. Notably, it has been demonstrated that Ca^2+^ entry through Ca^2+^ release activated Ca^2+^ channel (I_CRAC_) plays an essential role in mediating acidosis- and alkalinization-induced physiological/pathological functional changes. It was demonstrated that platelet stimulation results in cytoplasmic alkalinization and increased cytosolic Ca^2+^ concentration, which is essential for platelet aggregation in response to thrombin^11^. Similarly, extracellular acidosis-induced inhibition, as well as alkalosis-induced promotion of platelet aggregation is mediated by the changes of store-operated Ca^2+^ entry^12^. Moreover, store-operated Ca^2+^ entry was shown to mediate intracellular alkalinization in neutrophils^13^, and extracellular low pH was reported to inhibit I_CRAC_ in macrophages^14^. In Jurkat T-lymphocytes, cytosolic alkalinization induces Ca^2+^ release and Ca^2+^ entry^15^, and acidic internal and external pH inhibit I_CRAC_^16^. In SH-SY5Y neuroblastoma cells, however, store-operated Ca^2+^ entry was not affected by changes of intracellular pH, even though it was attenuated by low extracellular pH and potentiated by high extracellular pH^17^. In smooth muscle cells, extracellular acidosis decreases store operated Ca^2+^ entry, whereas extracellular alkalosis potentiates it^18^. Thus, it seems that changes of both intracellular and extracellular pH regulate I_CRAC_ activity or store-operated Ca^2+^ entry, albeit there are some discrepancies among different studies.

Since regulation of I_CRAC_ seems to play a critical role in acidosis- and alkalosis-associated physiological and pathological processes, it is essential to understand the molecular basis underlying pH regulation of I_CRAC_. As activation of I_CRAC_ requires coupling of Orai and STIM as well as gating of Orai^19^,^20^,^21^,^22^,^23^,^24^, alterations of either the coupling of Orai/STIM or gating properties of the pore-forming subunit Orai may cause functional changes of I_CRAC_. Indeed, it was demonstrated that intracellular low pH caused by oxidative stress induces uncoupling of Orai1 and STIM1, thereby inhibiting I_CRAC_^25^, and that intracellular high pH causes store depletion, thereby activating I_CRAC_. Moreover, mutation of the Ca^2+^ selective filter residue E106 in the channel pore (E106D) has been shown to alter acidic pH-dependent inhibition of I_CRAC_^26^. Furthermore, mutation of D110 and D112 (D110/112A) leads to reduced external pH sensitivity of Orai1/STIM1^27^. Whereas it is known that regulation of pore-forming subunit Orai1 by protons contributes to external pH sensitivity of Orai1/STIM1, the molecular mechanisms by which I_CRAC_ is regulated by internal pH is not fully understood.

Here we show that internal acidosis and alkalosis, as well as external acidosis and alkalosis markedly change Orai1/STIM1 channel functions. By investigating a series of mutants generated on residues located in the channel pore region, intracellular and extracellular loops, N- and C-termini, as well as transmembrane domains (TM3), we found that, in agreement with a previous report^26^, E106 is responsible for pH_o_ sensitivity when Ca^2+^ is the permeant cation. However, we found that E106 has no influence on pH_o_ sensitivity when Na^+^ is the charge carrier. Unexpectedly, we identified that the amino acid residue E190 located in TM3 of Orai1 is the major sensor of pH_o_ when Na^+^ is the charge carrier. Furthermore, we found that H155 located in the intracellular loop is responsible for intracellular pH sensitivity. Our results indicate that internal and external pH can regulate Orai1/STIM1 channel function by modulating the pore-forming subunit Orai1. Interestingly, our results suggest that the molecular basis for pH sensitivity when Ca^2+^ is the charge carrier is different from that of when Na^+^ is the charge carrier, an experimental condition which has been used for investigating pH regulation on Ca^2+^-selective and Ca^2+^-permeable channels. Thus, caution needs to be taken when extrapolating the mechanisms of pH sensitivity obtained using Na^+^ as the permeant cation to the physiological conditions when Ca^2+^ is the charge carrier. As E106, E190, and H115 are conserved residues in all the three isoforms of Orai, it is conceivable that they are the common external and internal pH sensors of different isoforms of Orai/STIM channels.

## Results

### Effects of extracellular pH on Orai1/STIM1 currents

**Figure 1:**
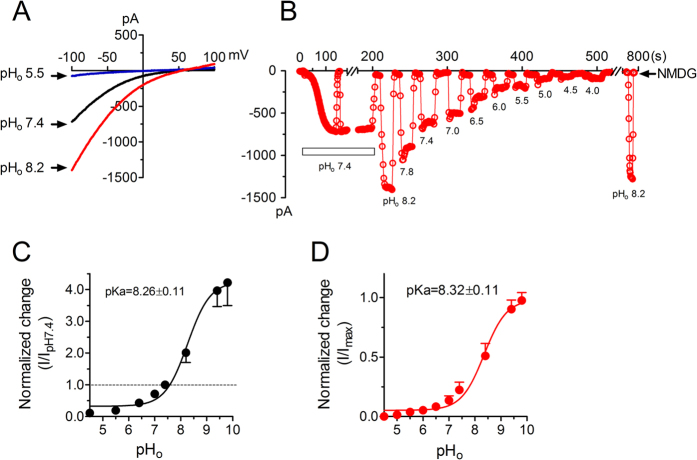
Effects of acidic and basic pH_o_ on Orai1/STIM1 channels heterologously expressed in HEK-293 cells. (**A**) Orai1/Stim1 currents elicited by a ramp protocol ranging from −100 to +100 mV at pH_o_ 7.4, 5.5 and 8.2. Note the significant increase by pH_o_ 8.2 and marked inhibition by pH_o_ 5.5. (**B**) Concentration-dependent effects of pH_o_ on Orai1/STIM1. Inward current was measured at −100 mV. The effects of pH_o_ was evaluated by perfusing the cells with different pH_o_ in DVF after activation of Orai1/STIM1 in pH_o_ 7.4 DVF, and NMDG solution was perfused to ensure that there was no leak current during the experiment. (**C**) Changes of current amplitude at each pH_o_ normalized to the current amplitude at pH_o_ 7.4. The best fit of the dose-response curve yielded pKa of 8.26 ± 0.11 (n = 8). (**D**) Dose-response curve analyzed by normalizing current amplitude at each pH_o_ to the maximal current amplitude. Best fit of the dose-response curve produced similar pKa (8.32 ± 0.11, n = 8) to that shown in (**C**).

Orai1/STIM1 currents were recorded by including high EGTA concentration in the pipette solution to passively induce store depletion. The effects of extracellular pH on Orai1/STIM1 were evaluated by perfusing the cells with external divalent free solutions (DVF) at various pHs after Orai1/STIM1 activation reached a steady-state. As shown in Fig. 1A, Orai1/STIM1 currents were elicited by a ramp protocol ranging from −100 to +100 mV in the DVF extracellular solutions. Current amplitude was significantly increased when the cell was exposed to high pH_o_. Without store depletion, Orai1/STIM1 currents were not able to be induced by high pH_o_, indicating that basic pH_o_ potentiates but does not activate Orai1/STIM1 channels (Fig. S1). In contrary to the effects of alkaline pH_o_, acidic pH_o_ markedly inhibited current amplitude. A concentration dependent effect of external pH on Orai1/STIM1 is shown in Fig. 1B. Current amplitude was enhanced 3- to 4-fold at pH_o_ 9, and was inhibited to a minimal level at pH_o_ 4.5. The effects of pH were reversible as shown in Fig. 1B. The changes of current amplitude at various pH normalized to the current amplitude at pH 7.4 are shown in Fig. 1C. The best fit of the dose-response curve yielded a pKa of 8.26 ± 0.11 (Fig. 1C). Similar pKa (8.32 ± 0.11) was also obtained by the best fit of the normalized currents in reference to the maximal current amplitude (Fig. 1D).

**Figure 2 f2:**
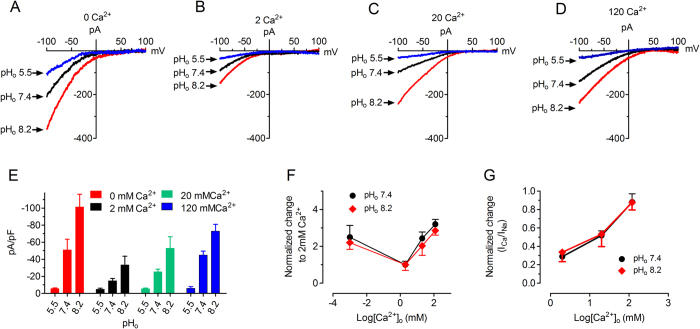
Effects of pH_o_ on Orai1/STIM1 at various extracellular Ca^2+^ concentrations. (**A**–**D**) Representative currents recorded at pH_o_ 5.5, 7.4 and 8.2 with extracellular Ca^2+^ concentrations of 0, 2, 20 and 120 mM. E, Averaged current amplitude measured at −100 mV at pH_o_ 5.5, 7.4, and 8.2 in various extracellular Ca^2+^ concentrations. Acidic pH_o_ 5.5 significantly inhibited current amplitude (p < 0.01; n = 8 ~ 17) and basic pH_o_ 8.2 remarkably potentiated current amplitude at each extracellular Ca^2+^ concentration (p < 0.01; n = 8 ~ 17). F, Normalized current amplitude at each Ca^2+^ concentration to the current amplitude at 2 mM Ca^2+^. G, Normalized Ca^2+^ current amplitude at each Ca^2+^ concentration to the Na^+^ current amplitude at 0 mM Ca^2+^. Analysis with two-way ANOVA indicated that the effects of pH_o_ 8.2 on Orai1/STIM1 was independent of extracellular Ca^2+^ concentration (**F**,**G**).

The results shown in Fig. 1 were obtained using DVF solution because DVF solution produces larger current amplitude. We next tested the effects of various pHs on Orai1/STIM1 currents recorded in Tyrode’s solutions containing 2, 20 and 120 mM Ca^2+^, respectively. As show in Fig. 2A–D, pH_o_ 5.5 inhibited and pH_o_ 8.2 enhanced Orai1/STIM1 inward current independent of extracellular Ca^2+^ concentrations. In non-transfected control cells, high or low pH_o_ did not induce any current (Fig. S2). The averaged current amplitude under different conditions is shown in Fig. 2E. Two-way ANOVA analysis indicated that the effect of pH_o_ on Orai1/STIM1 was independent of extracellular Ca^2+^ concentrations. The ratios of the current amplitude at various extracellular Ca^2+^ concentrations versus the current amplitude recorded in 2 mM Ca^2+^ Tyrode solution at pH_o_ 8.2 were similar to those at pH_o_ 7.4 (Fig. 2F), suggesting that potentiation of Orai1/STIM1 by basic pH was not significantly influenced by extracellular Ca^2+^ concentrations. Similarly, the normalized ratios of Ca^2+^ current at different Ca^2+^ concentrations versus Na^+^ current in DVF at pH_o_ 8.2 are well superimposed with the ratios at pH_o_ 7.4 (Fig. 2G), further suggesting that modulation of Orai1/STIM1 channel activity by protons is not dependent on the charge carrier. Thus, we first used DVF extracellular solution to investigate the effects of pH_o_ on Orai1/STIM1 channels.

### Effects of external pH on Orai2/STIM1 and Orai3/STIM1 currents

**Figure 3 f3:**
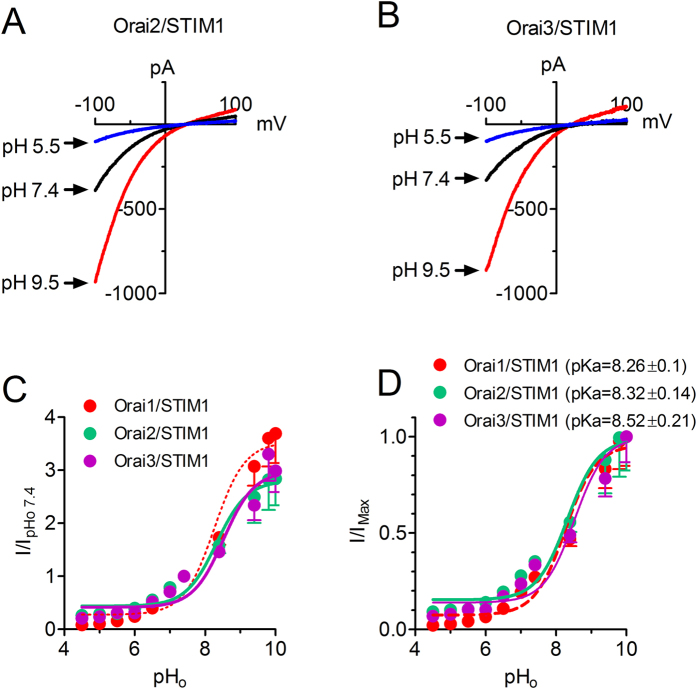
Effects of extracellular protons on Orai2/STIM1 and Orai3/STIM1. (**A,B**) Representative recordings at pH_o_ 5.5, 7.4 and 9.5. (**C**) Concentration-dependent effects of external protons on Orai2/STIM1 and Orai3/STIM1 in comparison with Orai1/STIM1. Current amplitude was normalized to that at pH_o_ 7.4. (**D**) Dose-response curve obtained by normalizing to maximal current amplitude. Best-fit of the dose-response curves yielded pKa of 8.32 ± 0.14 for Orai2/STIM1 and 8.52 ± 0.21 for Orai3/STIM1, identical to the pKa obtained in (**C**). Note that the dashed lines in (**C,D**) represent the data of Orai1/STIM1 taken from Fig. 1C,D.

Before we went on to investigate the molecular mechanism of pH_o_ regulation on Orai1/STIM1, we tested if external pH regulates channel activity of Orai2/STIM1 and Orai3/STIM1. As shown in Fig. 3, Orai2/STIM1 and Orai3/STIM1 currents were significantly potentiated by basic pH_o_ and inhibited by acidic pH_o_ (Fig. 3A,B). The fold changes of current amplitude by normalizing current amplitude at each pH to that of pH_o_ 7.4 are shown in Fig. 3C. The maximal increases in Orai2/STIM1 and Orai3/Sim1 at high pH are about 3 fold, similar to the maximal increase of Orai1. The dose-response curves obtained by normalizing current amplitude at each pH to the maximal current amplitude are shown in Fig. 3D. The dose-response curves of Orai1/STIM1, Orai2/STIM1, and Orai3/STIM1 are well superimposed. The pKa obtained from the best fit of the dose-response curves are 8.32 ± 0.14 and 8.52 ± 0.21 for Orai2/STIM1 and Orai3/STIM1 respectively, similar to the pKa of Orai1/STIM1 shown in Fig. 1 (dotted lines in Fig. 3). These results indicate that Orai1/STIM1, Orai2/STIM1 and Orai3/STIM1 have similar pH_o_ sensitivity.

### Mechanisms of external pH regulation on Orai1/STIM1

**Figure 4 f4:**
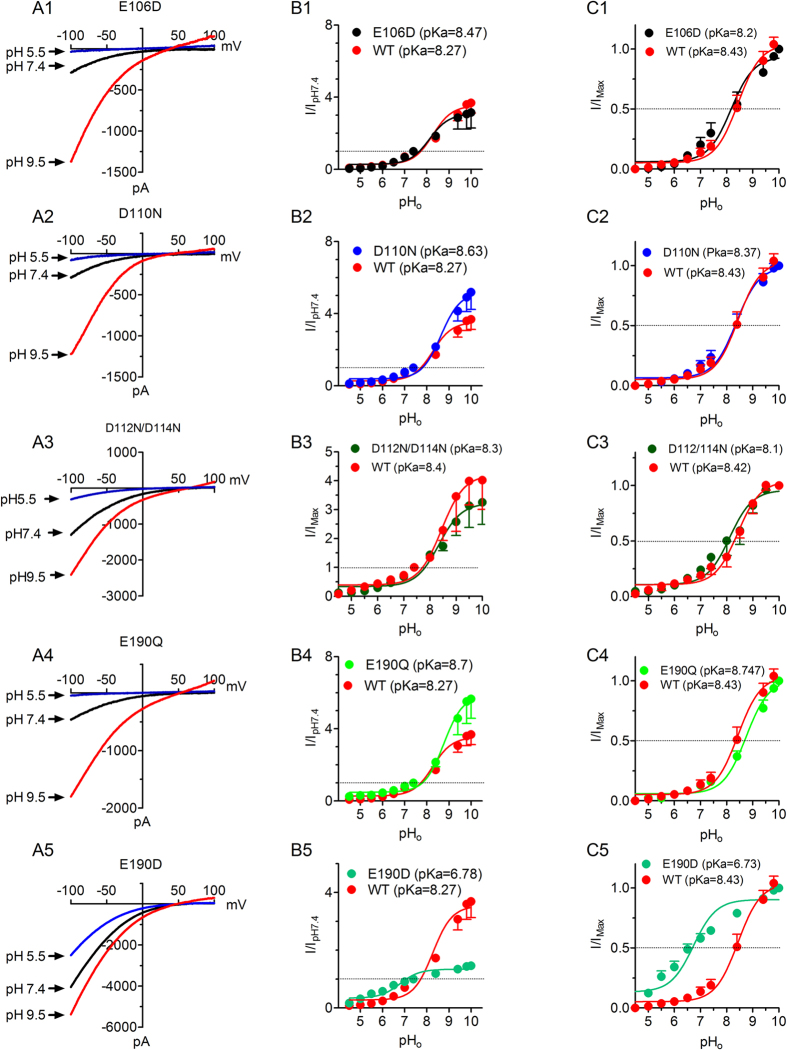
Concentration-dependent effects of protons on Orai1/STIM1 WT channel and mutants. (**A1**–**A5**), Typical recordings of each mutant at pH_o_ 5.5, 7.4 and 9.5. (**B1**–**B5**) Concentration-dependent effects of various pHs on current amplitude of each mutant in comparison with WT. The changes of current amplitude were obtained by normalizing current amplitude at each pH_o_ to the value at pH_o_ 7.4. The pKa of each mutant was obtained by best fit of the dose-response curves. (**C1**–**C5**) Dose-response curves constructed using the normalized current amplitude to the maximal current amplitude. The best fit of the dose-response curves generated pKa of 8.43 ± 0.1 (n = 8) for WT, 8.17 ± 0.13 (n = 9, p > 0.05) for E106D, 8.37 ± 0.09 (p > 0.05, n = 8) for D110N, 8.10 ± 0.18 (n = 12, p > 0.05) for D112/114N, 8.74 ± 0.08 (n = 4 ~ 6, P > 0.05) for E190Q, and 6.73 ± 0.08 (n = 8, p < 0.05) for E190D.

Activation of Orai1/STIM1 involves coupling of Orai1 and STIM1 as well as gating of Orai1. Since the external pH enhanced Orai1/STIM1 current amplitude after the channel was fully activated, we reasoned that protons may directly modulate the pore-forming subunit Orai1. To understand the mechanism by which external protons regulate Orai1/STIM1, we generated mutations by neutralizing a series of negatively charged residues located on the external site of the channel or along the channel pore, including E106Q, D110N, D112/114N, and E190Q. We also generated the mutations E106D and E190D. The negatively charged residues E106, D110, and E190 are conserved residues in all three isoforms of Orai, whereas the negatively charged residues of D112 and D114 are only conserved in Orai1 and Orai3 (Fig. S3). The mutant E106Q produced minimal current, consistent with the inability of E106Q to produce Ca^2+^ influx^20^ and the dominant-negative effects of E106Q reported previously^28^,^29^. Thus, we did not investigate E106Q in detail. For all the other mutants, representative recordings at pH_o_ 5.5, 7.4 and 9.5 are shown in Fig. 4A1–A5. Changes of current amplitude at each pH_o_ are shown in Fig. 4B1–B5, and the dose-response curves obtained by normalizing current amplitude at each pH_o_ to the maximal current amplitude are shown in Fig. 4C1–C5. Whereas the majority of mutants were inhibited by pH_o_ 5.5 and enhanced by pH_o_ 8.4 to a similar degree in comparison to WT, the mutant E190D displayed much smaller inhibition at pH_o_ 5.5 and significantly reduced potentiation at pH_o_ ≥8.4 in comparison to WT Orai1/STIM1 channels (Fig. 4B1–B5, [Fig f2], [Fig f3], [Fig f4], [Fig f5],C1–C5). In fact, E190D largely diminished the sensitivity to both acidic pH_o_ and alkaline pH_o_, whereas E106D displayed similar pH sensitivity to WT Orai1/STIM1 (Fig. 4A1–C1, and A5–C5). The pKa of each mutant obtained by the best fit of the concentration-dependent changes of current amplitude (Fig. 4B1-B5) is similar or identical to the pKa obtained by the best fit of the normalized dose-response curve (Fig. 4C1–C5). The dose-response curves of E106D, D110N, and D112N/D114N were well superimposed with that of WT Orai1/STIM1 (Fig. 4C1–C3; p > 0.05). At much higher pH_o_ such as pH_o_ 9.0, 9.4 and 10.0, the potentiation of D110N was greater than that of WT but did not reach statistical significance (Fig. 4B2), and the potentiation of D112N/D114N was smaller than that of WT (Fig. 4B3) without reaching statistical differences. The dose-response curve of E190Q was slightly right-shifted without causing significant changes in pKa (p > 0.05; Fig. 4C4), albeit the increase of E190Q current amplitude by high pH_o_ was greater than that of WT Orai1/STIM1 (Fig. 4B4). By contrast, the dose-response curve of E190D was significantly shifted to the left, resulting in a pKa that is almost two pH units lower than that of WT Orai1/STIM1 (p < 0.05; Fig. 4C5). The significant change in pKa in the E190D indicates that E190 is essential for the extracellular pH sensitivity of Orai1/STIM1 channels in DVF solution. Since E190Q only generated a small change in pKa, whereas E190D shifted the dose-response curve significantly, it is likely that the length of the side chain rather than the charge of E190 plays a key role in pH_o_ modulation of Orai1/STIM1. Taken together, although D110N and D112N/D114N produce some changes in potentiation of current amplitude at very high pH_o_, it seems that the conserved residue E190 plays a major role in pH_o_ sensitivity of Orai1/STIM1 when Na^+^ is the charge carrier.

### Effects of internal pH_i_ on Orai1/STIM1 channels

**Figure 5 f5:**
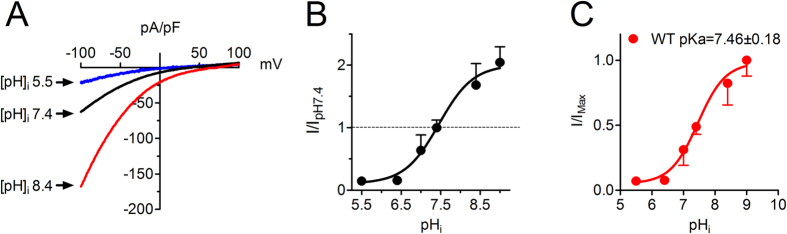
Effects of internal pH on Orai1/STIM1. (**A**) Representative Orai1/Stm1 currents elicited by a ramp protocol ranging from −100 to +100 mV in cells with pipette solution at pH_i_ 5.5, 7.4 and 8.4, respectively. (**B**) Concentration-dependent effects of pH_i_ on Orai1/STIM1 currents. Current amplitude measured at −100 mV at each pH_i_ was normalized to the current amplitude at pH_i_ 7.4. Current amplitude was almost completely inhibited at pH_i_ 5, and was enhanced by about 2-fold at pH_i_ 9 (n = 12, 5, 6, 44, and 6 at pH_i_ 5.5, 6.5, 7.4, 8.4, and 9, respectively). (**C**) Dose-response curve analyzed by normalizing current amplitude at each pH_i_ to the maximal current amplitude at pH_i_ 9. Best fit of the dose-response curve produced same pKa (7.46 ± 0.18, n = 12, 5, 6, 44 at pH_i_ 5.5, 6.5, 7.4, 8.4, and 9, respectively).

To study whether internal pH (pH_i_) also influences Orai1/STIM1 channel activity, we titrated the pipette solution at various pHs. At acidic pH_i_ 5.5, Orai1/STIM1 current amplitude was only about 10% of the current amplitude at pH_i_ 7.4 (Fig. 5A), whereas at basic pH_i_ 8.4, the current amplitude was almost two-fold greater than the current amplitude at pH_i_ 7.4 (Fig. 5A). The internal pH regulates Orai1/STIM1 channel activity in a concentration-dependent manner (Fig. 5B), with the maximal increase of current amplitude by 2-fold at pH_i_ 9 (Fig. 5B). The best fit of the dose-response curve yielded pKa of 7.46 (Fig. 5C). Thus, similar to the effects of pH_o_ on Orai1/STIM1, internal acidic pH inhibits and alkaline pH potentiates Orai1/STIM1 channel activities.

### Molecular mechanisms of internal pH sensitivity of Orai1/STIM1

**Figure 6:**
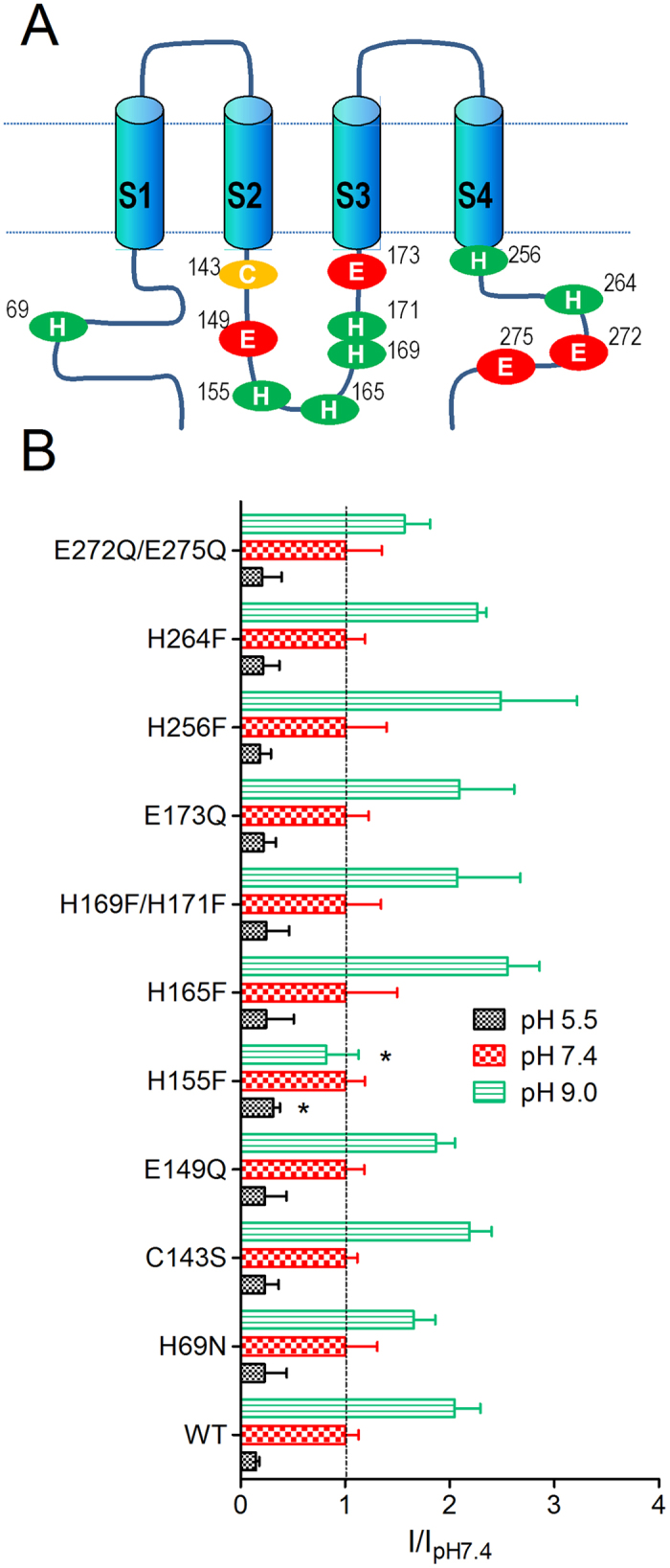
Effects of neutralization of internal residues on pH_i_ sensitivity of Orai1/STIM1. (**A**) Schematic diagram of Orai1 showing the position of the substituted residues. There were 8 single mutants and two double mutants. (**B**) Averaged changes of current amplitude by pH_i_ 5.5 and 9.0 in the mutants in comparison with the WT Orai1/STIM1. Current amplitude at pH_i_ 5.5 and 9.0 was normalized to that at pH_i_ 7.4. Data were analyzed by ANOVA followed by t-test with Bonferroni correction.

To understand the mechanism by which Orai1/STIM1 channels are regulated by pH_i_ changes, we made a series of mutations at the N- and C- termini, and at the loop between TM2 and TM3 (Fig. 6A). The mutations were made on the titratable residues including histidine (His) and glutamic acid (Glu), as well as cysteine (Cys) residues which have been shown to be involved in internal pH sensing in other channels^30^,^31^,^32^. We first tested current amplitude of each mutant at pH_i_ 5.5 and 9.0 in comparison with the current amplitude at pH_i_ 7.4. Representative recordings at pH_i_ 5.5, 7.4, and 9.0 of each mutant are shown in Fig. S4. The normalized current amplitude of each single or double mutant is shown in Fig. 6B. Similar to WT Orai1/STIM1, current amplitude of all the mutants was markedly inhibited at pH_i_ 5.5 and significantly enhanced at pH_i_ 9.0 except for H155F. For H155F, current amplitude at pH_i_ 9.0 was even smaller than that at pH_i_ 7.4, whereas the current amplitude of other mutants was enhanced 1.5- to 2-fold at pH_i_ 9.0, which was similar to the changes in WT Orai1/STIM1. At pH_i_ 5.5, current amplitude of H155F was significantly larger than WT (p < 0.05). These results indicate that H155F is less sensitive to both acidic and basic internal pH.

**Figure 7:**
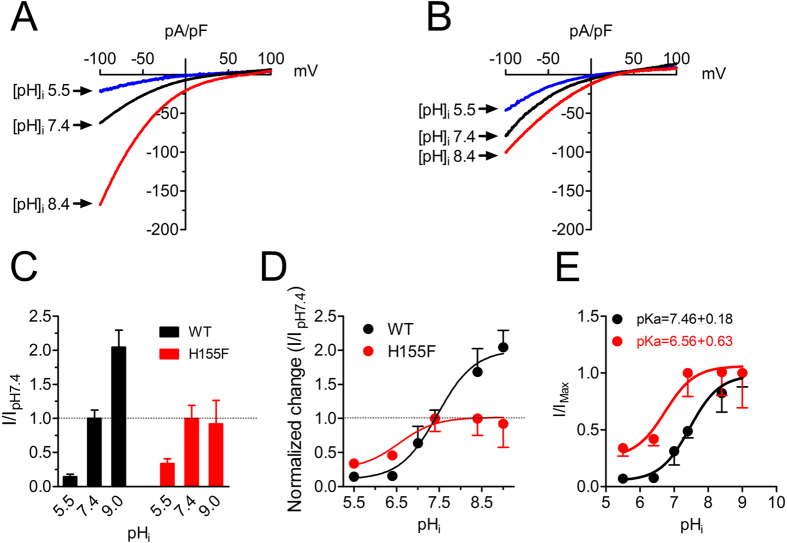
H155 is the intracellular pH sensor of Orai1/STIM1 channels. (**A**,**B**) effects of pHi 5.5 and pHi 8.4 on H155F in comparison with WT Orai1/STIM1. The original recordings of WT Orai1/STIM1 in A are from the Fig. 5A. Note that pH_i_ 5.5 and pH_i_ 8.4 produced much smaller change on H155F (**B**) compared with WT Orai1/STIM1 (**A**). (**C**) Averaged changes of current amplitude by pH_i_ 5.5 and 9.0. (**D**) Concentration-dependent effects of pH_i_ on H155F compared with WT Orai1/STIM1. Note that alkaline pH_i_ failed to potentiate H155F current amplitude, whereas acidic pH_i_ produced much smaller inhibition in comparison with WT Orai1/STIM1. E, Dose-response curves constructed using current amplitude normalized to the maximal current amplitude. The pKa was 7.46 ± 0.18 (n = 6–44) and 6.56 ± 0.63 (n = 6–11) for WT and H155F mutant.

We further investigated the effects of different pH_i_ on H155F in comparison with WT Orai1/STIM1. Original recordings of WT and H155F at pH_i_ 5.5, 7.4, and 8.4 are shown in Fig. 7A,B, and the average current amplitude is shown in Fig. 7C. The concentration-dependent changes of H155F current amplitude normalized to I_pHi 7.4_ are shown in Fig. 7D. It is noticeable that H155F lost response to basic pH_i_, and the response to acidic pH_i_ was also significantly diminished. The pKa obtained by best fit of the dose-response curves constructed using I/I_Max_ was 6.56 ± 0.63 for H155F and 7.47 ± 0.18 for WT Orai1/STIM1 (Fig. 7E). The dose-response curve of H155F was left-shifted by almost one pH unit (p < 0.01). These results suggest that H155 is responsible for intracellular pH sensitivity of Orai1/STIM1.

### Endogenous I_CRAC_ channel exhibits similar pH sensitivity to Orai1/STIM1

**Figure 8:**
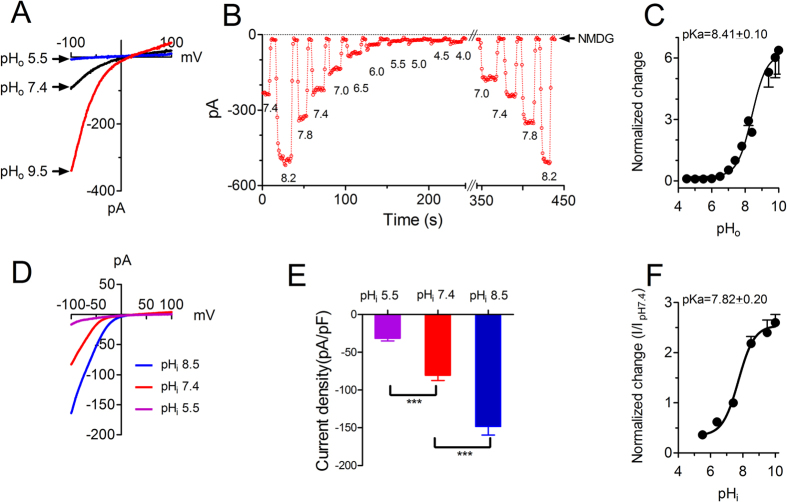
Effects of pH_o_ and pH_i_ on endogenous I_CRAC_ currents in RBL cells. (**A**) Representative recording of I_CRAC_ in RBL cells. Currents were elicited by a ramp protocol ranging from −100 to +100 mV in DVF solutions. After I_CRAC_ activation reached a steady-state, the cell was exposed to external solutions with different pH. (**B**) Concentration-dependent effects of pH_o_ on I_CRAC_. NMDG was used to test leak current. The inhibition and potentiation effects of pH_o_ were reversible. (**C**) Dose-response curve constructed using current normalized to the current amplitude at pH_o_ 7.4. The best fit of the dose-response curve yielded pKa of 8.41 ± 0.10 (Mean ± SEM, n = 8–11). (**D**) Representative traces of I_CRAC_ at pH_i_ 5.5, 7.4, and 8.5. (**E**) Average current amplitude of I_CRAC_ at pH_i_ 5.5, 7.4 and 8.5. (**F**) Dose-response curve constructed using current normalized to the current amplitude at pH_i_ 7.4. The best fit of the dose-response curve yielded pKa of 7.82 ± 0.2 (Mean ± SEM, n = 7–13).

To investigate whether endogenous I_CRAC_ channels are also sensitive to pH changes, we used RBL cells for current recording in DVF solution under various pH_o_ conditions. I_CRAC_ was activated by passive store depletion, and then cells were perfused with different external pH solutions. As shown in Fig. 8A, I_CRAC_ was completely inhibited by pH_o_ 5.5, and significantly increased by pH_o_ 9.5. The effect of pH_o_ on I_CRAC_ was concentration dependent and reversible (Fig. 8B). The pKa of I_CRAC_ was 8.41 ± 0.10, similar to the pKa of Orai1/STIM1 shown in Fig. 1. Moreover, the I_CRAC_ current in RBL cells was also regulated by internal acidic and alkaline pH. Current amplitude was inhibited by about 80% at pH_i_ 5.5, and potentiated by about 1.5–2 fold at pH_i_ 8.5 (Fig. 8D,E). The pKa (Fig. 8F) of I_CRAC_ was also similar to that of Orai1/STIM1 shown in Fig. 7D.

### Effects of external Ca^2+^ on pH sensitivity of Orai1/STIM1 mutants

**Figure 9:**
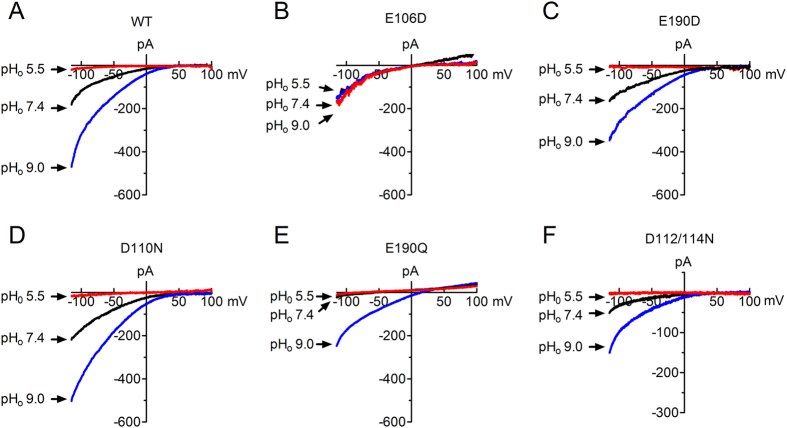
Effects of external pH on Orai1/STIM1 channel and its mutants in external solutions with 20 mM Ca^2+^. (**A**–**F**) Original recordings in 20 mM Ca^2+^ Tyrode’s solution at pH_o_ 5.5, 7.4 and 9.0. Please note that E106D, especially E190Q, conducted very small currents. E106D is insensitive to both pH_o_ 5.5 and pH_o_ 9.0, whereas E190Q is insensitive to pH_o_ 5.5.

DVF solution has been commonly used for investigating pH sensitivity of different ion channels^33^,^34^,^35^,^36^,^37^,^38^,^39^. Although the pH sensitivity of WT Orai1/STIM1 is not dependent on charge carrier (Fig. 2), we tested pH sensitivity of the Orai1 mutants when Ca^2+^ is the permeant cation for Orai1/STIM1 channels, since changing the relative permeability and/or selectivity may serve as a mechanism by which protons regulate Ca^2+^-permeable channels^35^,^40^,^41^ including I_CRAC_^26^,^27^. We used Ca^2+^ concentration (20 mM) higher than physiological Ca^2+^ (2mM) in order to get larger currents. Similarly high Ca^2+^ concentration for I_CRAC_ recording has been used previously^26^,^27^. As shown in Fig. 9, in the presence of 20 mM external Ca^2+^, the mutants D110N and D112/114N displayed similar pH sensitivity to that of WT Orai1/STIM1, indicating that the residues D110 and D112/114 do not contribute to pH sensitivity of Orai1/STIM1, which is consistent with the results obtained in DVF solution (Fig. 4). However, the mutant E106D was insensitive to pH changes in 20 mM Ca^2+^ Tyrode’s solution, a feature which presumably manifests the reduced Ca^2+^ permeability and selectivity as previously reported^26^. Similarly, the mutant E190Q, which largely loses Ca^2+^ selectivity^20^, conducted very minimal currents and was insensitive to low pH (Fig. 9E), albeit a large inward current was elicited at pH 9.0 with an unknown mechanism which is worthy of further investigation in the future. Interestingly, the mutant E190D has similar pH sensitivity as the WT Orai1/STIM1 in 20 mM Ca^2+^ solution (Fig. 9F). This is not surprising given that E190D preserves similar Ca^2+^ selectivity to that of WT Orai1/STIM1 channels^20^.

Using normal Tyrode’s solution containing 2 mM Ca^2+^ as the external solution, we also tested whether external Ca^2+^ influences pH sensitivity of the internal pH sensor of Orai1/STIM1, H155F. We found that the pH sensitivity of H155F was not altered by external Ca^2+^ concentration. For example, the ratios of current amplitude at pH_i_ 5.5 and pH_i_ 8.4 versus pH_i_ 7.4 were 0.34 ± 0.07 (n = 6) and 1.1 ± 0.3 (n = 6) respectively in 2 mM Ca^2+^ Tyrode’s solution, similar to the ratios of 0.36 ± 0.05 and 1.0 ± 0.24 (n = 6) obtained in DVF solution as shown in Fig. 7.

## Discussion

In this study, we demonstrate that Orai1/STIM1 channels are regulated by both internal and external pH. Whereas acidic internal and external pH inhibit channel activity, alkaline intra- and extracellular pH dramatically increase channel activity. We identify a new residue H155 which is responsible for intracellular acidic and alkaline pH sensitivity. For extracellular pH sensitivity, we find that even though pH regulation on Orai1/STIM1 channels is independent of the charge carrier, the mechanisms underlying pH sensitivity are different with different charge carriers. While the residue E106 is responsible for the pH sensitivity when Ca^2+^ is the charge carrier, we find that E190 is responsible for pH sensitivity when Na^+^ is the charge carrier. Furthermore, we show that endogenous I_CRAC_ in RBL cells is also inhibited by acidic pH_i_ and pH_o_, and potentiated by basic pH_i_ and pH_o_. As I_CRAC_ plays an important role in various physiological functions, and given that acidosis and alkalinization are involved in a variety of physiological/pathological processes, I_CRAC_ may play a crucial role in mediating various physiological/pathological functions associated with acidosis and alkalinization.

### Orai1/STIM1 channel activity is potentiated by intracellular and extracellular alkalinization, and inhibited by internal and external acidosis

Regulation of the endogenous I_CRAC_ by acidic pH in Jurkat T-lymphocytes and by external alkaline pH in macrophages has been previously reported^14^,^16^. Moreover, two recent studies have shown that heterologously expressed Orai1/STIM1 currents can be inhibited by external low pH^26^,^27^ and internal low pH^27^, and enhanced by external high pH but not by internal high pH^27^. We find that Orai1/STIM1 channels expressed in HEK-293 cells are not only inhibited by intracellular and extracellular acidic pH, but also enhanced by both intracellular and extracellular alkaline pH. The reason for the different regulation by internal high pH obtained in this study and in Beck’s study is currently unknown, and will need further investigation. However, previous studies demonstrated that in Jurkat T-lymphocytes and neutrophils, cytosolic alkalinization induces Ca^2+^ release and store-operated Ca^2+^ entry^13^,^15^, consistent with the notion that alkaline internal pH potentiates Orai1/STIM1. Similar to the regulation of over-expressed I_CRAC_ currents, the native I_CRAC_ is inhibited by external low pH and enhanced by high pH, with the pKa of 8.4 (Fig. 8). We show that pH regulates Orai1/STIM1 channel activities independent of charge carriers. For example, acidic pH_o_ inhibits I_CRAC_ currents carried by Ca^2+^ ions to the same degree as it inhibits the I_CRAC_ currents carried by Na^+^ ions. Likewise, basic pH_o_ enhances I_CRAC_ currents carried by both Ca^2+^ ions and Na^+^ ions (Fig. 2). Similar results have been reported for the regulation of native I_CRAC_ currents by pH^16^. Kerschbaum and colleagues demonstrated that I_CRAC_ currents in Jurkat T lymphocytes are inhibited by acidic extracellular and intracellular pH, and that both Ca^2+^ currents and monovalent Na^+^ currents of I_CRAC_ can be equally inhibited by acidic pH in a voltage independent manner^16^. The authors showed that acidic intracellular pH blocks I_CRAC_ with a pKa of 6.8, whereas acidic extracellular pH inhibits I_CRAC_ with a pKa of 8.2^16^. Alkaline external pH was also previously shown to enhance I_CRAC_ currents in macrophages with a pK_a_ of 8.2^14^. Our pKa for the external proton effects on Orai1/STIM1 is similar to that previously reported^14^,^16^, but our pKa (7.46) for internal pH effects is higher than that obtained by Kerschbaum and colleagues^16^. We do not yet know the reason for the different results, but it is conceivable that this discrepancy could be due to the fact that we used heterologously expressed Orai1/STIM1 channels, whereas the native I_CRAC_ may be contributed by different isoforms of Orai and STIM^42^. Nonetheless, regulation of endogenous I_CRAC_ and heterologously expressed Orai/STIM currents by pH indicates that I_CRAC_ may be an important mediator of altered Ca^2+^ signaling under acidic and alkaline conditions.

### Mechanisms of external pH sensitivity of Orai1/STIM1 channels

By mutating a series of titratable amino acid residues located in the channel pore, in the loop between TM1 and TM2, and within TM3^43^,^44^, we find that the amino acid residues which are responsible for extracellular pH sensitivity are different with different permeant cations. Whereas E106D loses pH sensitivity when Ca^2+^ is the charge carrier, which is consistent with the previous report^26^, E106D shows similar pH sensitivity to that of WT Orai1/STIM1 in the absence of Ca^2+^ (in DVF solution) when Na^+^ is the charge carrier. E190D, however, exhibits markedly reduced pH sensitivity in DVF solution when Na^+^ is the permeant cation, albeit displaying similar pH sensitivity to that of WT Orai1/STIM1 in the Tyrode’s solution containing Ca^2+^. Thus, it appears that E190 is responsible for external pH sensitivity in the absence of Ca^2+^ when Na^+^ is the charge carrier, whereas E106 is responsible for external pH sensitivity when Ca^2+^ is the charge carrier. Although using monovalent Na^+^ cation in DVF solution as the charge carrier is a non-physiological condition, Na^+^ in DVF solution has been commonly used as the permeant cations for investigating pH sensitivity of different ion channels including Ca^2+^ channels and Ca^2+^-permeable channels^33^,^34^,^35^,^36^,^37^,^38^,^39^. Thus, our results provide important insights suggesting that the molecular basis for pH sensitivity can be different when using Ca^2+^ as the charge carrier or the monovalent cation Na^+^ as the charge carrier, and that precautions need to be taken when extrapolating the mechanism of pH sensitivity obtained from experimental conditions to physiological conditions.

External protons can regulate ion channel functions via changing gating properties and/or influencing channel permeation^33^,^34^,^35^,^36^,^40^. Many Ca^2+^ permeable channels are regulated by protons via changing Ca^2+^ selectivity and permeability. For the voltage-gated Ca^2+^ channels (VGCC), the Ca^2+^ selectivity and pH sensitivity are conferred by the Glu residues in the pore-forming region^35^,^36^. Mutation of Glu by glutamine (Gln) substitution in repeats I or III produces similar low conductance single channel currents, mimicking the protonated state^35^,^36^. Thus, the permeant divalent cations and protons compete for the same Glu binding sites^35^. The similar competing mechanism also underlies pH modulation on TRPM6 and TRPM7 channels^41^,^45^,^46^. Yet, for TRPM6 and TRPM7 channels, binding of protons to the E1024 (TRPM6) and E1047 (TRPM7) in the channel pore releases Ca^2+^ block on monovalent currents. Therefore, low pH_o_ potentiates TRPM6 and TRPM7 currents by enhancing the monovalent current amplitude^41^. Similar to VGCCs, the Ca^2+^ selective TRPV5 channels are inhibited by acidic pH_o_^34^. However, the pH_o_ sensing site E522 is independent of the Ca^2+^ selectivity site D648^34^. Protons modulate TRPV1 channel activity via influencing both gating and permeation properties^37^,^38^,^39^,^40^. Activation of TRPV1 by protons is mediated by the extracellular E600 and E648 residues^37^, whereas the divalent permeability of TRPV1 is conferred by D646^47^. TRPM2 has also been shown to be inhibited by external protons^48^,^49^,^50^,^51^, yet the mechanism is more complicated^48^,^49^,^50^. Although one study suggested that external protons permeate through TRPM2 and inhibit channel activity intracellularly^49^, mutagenesis results in other studies demonstrated that external protons bind to external residues around the channel pore and block the channel activity extracellularly^48^,^50^,^51^. Whereas neutralization of H958, D964, and E994 at the outer vestibule of the channel pore enhances pH sensitivity of TRPM2 by reducing external Ca^2+^ sensitivity^48^, Yang and colleagues demonstrated that external protons inhibit TRPM2 in a voltage- and state-dependent manner^50^,^51^, and that substitution of the residues K952 and D1002 by alanine (Ala) significantly reduces inhibition of open TRPM2 channels by external protons^50^. Furthermore, the residue Q992 in the outer pore of mouse TRPM2 (mTRPM2) is crucial for the reduced sensitivity to pH_o_ 6.0 in comparison with human TRPM2 (hTRPM2)^51^. Nonetheless, it appears that the pH sensing residues are usually located either in the channel pore, or at the vestibule of the channel pore for many Ca^2+^-selective and Ca^2+^-permeable channels^39^. For Orai1, however, we found that neutralizing the negatively charged residues D110, D112 and D114 at the vestibule did not significantly change pH_o_ sensitivity regardless of permeant cations (Figs 4 and 9). At very high pH_o_ (pH_o_ 9.0, 9.4, and 10.0), D110N and D112/114N exhibited larger current than WT but without statistical significance (Fig. 4) when Na^+^ is the charge carrier. Interestingly, when Ca^2+^ is the permeant cation, substitution of the Ca^2+^ selective filter residue E106 by Asp (E106D) eliminates pH_o_ sensitivity, presumably through changes of Ca^2+^ permeability and selectivity, a similar mechanism by which VGCCs and some other Ca-permeable channels are regulated by protons, as previously reported^26^. However, when Na^+^ is the permeant cation, E106D did not produce any change in pH sensitivity, and the pH dose-response curve of E106D was well superimposed with that of WT channels. Unexpectedly, we found that when Na^+^ is the permeant cation, mutant E190D significantly altered the pH sensitivity of Orai1/STIM1, and shifted the pH dose-response curve by almost two pH units.

How might E190 influence pH sensitivity of Orai1/STIM1 channels when Na^+^ is the charge carrier? The E190 residue in the TM3 is not facing the channel pore or involved in the pore formation^43^,^52^, even though E190, W176 and G183 in the TM3 have been shown to be involved in Ca^2+^ selectivity, channel gating and fast inactivation^53^,^54^. Since E190 is not facing the channel pore, the mechanisms by which E190 affects both Ca^2+^ selectivity and/or the size of channel pore are not understood from a structural point of view^55^. Amcheslavsky and colleagues recently demonstrated that E165 in Orai3, the residue equivalent to E190 in Orai1, is involved in 2-APB induced gating of Orai3 channels^55^. The authors proposed that E165 of TM3 is directly behind and in between two TM1 domains, and when 2-APB activates Orai3, E165 moves toward the central axis of the channel, therefore affecting channel permeation and pore formation^55^. Similarly, it is conceivable that E190 in Orai1 becomes accessible when the channel opens. Since E190Q did not significantly change the pH_o_ sensitivity, whereas E190D exhibited markedly reduced sensitivity to both acidic and basic pH_o_ resulting in a significant shift of the pH_o_ dose-response curve by almost two pH units, it is likely that the side chain but not the charge of E190 plays an essential role in pH_o_ sensitivity. Thus, a plausible model is that when Orai1/STIM1 channel opens, E190 becomes accessible to the channel pore. Changing the length of the side chain of E190 may alter the size of the ion-permeation pathway, thereby influencing ion permeation when Na^+^ is the permeant cation, resulting in changed pH sensitivity. Nonetheless, further investigation such as mutating E190 to different residues, measuring single channel conductance by noise analysis, and evaluating changes of permeability will provide more evidence in order to fully understand the underlying mechanisms.

In agreement with our result that mutant E106D eliminates pH sensitivity at high and low pH (Fig. 9) when Ca^2+^ is the permeant cation, Scrimgeour and colleagues showed that E106D changes Ca^2+^ selectivity as well as fast Ca^2+^-dependent inactivation^44^,^45^, and eliminates acidic pH inhibition of Orai1/STIM1^26^. The authors demonstrated that acidic pH inhibits Orai1/STIM1 with a pKa of 7.8^26^. Similar to our results, the authors described that Na^+^ conductance of the WT Orai1/STIM1 channels in the presence and absence of external Ca^2+^ displayed the same pH dependence^26^, though whether E106 is responsible for the pH sensitivity in the absence of Ca^2+^ was not investigated^26^. Since E106D alters Ca^2+^ selectivity and Ca^2+^ affinity (25 μM for WT and 490 μM for E106D)^53^, we believe that the changes in pH sensitivity in E106D is caused by altered Ca^2+^ selectivity and permeability as previously demonstrated^26^. Indeed, when we used Na^+^ as the charge carrier, E106D is no longer sensitive to high and low pH_o_. Instead, we found that in the absence of Ca^2+^, mutant E190D markedly changed pH_o_ sensitivity in comparison with the WT Orai1/STIM1 channels. Thus, our results indicate that E190 is the pH_o_ sensor of Orai1/STIM1 when Na^+^ is the permeant cation.

Although we found that the mutants D110N and D112/114N did not significantly alter pH_o_ sensitivity, a recent study by Beck and colleagues demonstrated that D110/D112A shows reduced sensitivity to external pH 8.4 and pH 6.0^27^. As we neutralized the aspartic acid (Asp) at D110 and D112 to asparagine (Asn), whereas Beck and colleagues mutated Asp residues to Ala residues, it is plausible that the size of the residue at D110 and D112 plays a role in sensing external pH. Alternatively, mutating D110 and D112 simultaneously (D110/D112A) may be crucial for altering pH_o_ sensitivity, which may explain the discrepancy between our and their results. However, since Orai2/STIM1 and Orai3/STIM1 display similar pH_o_ sensitivity to that of Orai1/STIM1 (Fig. 3), and the negatively charged residue D112 is not conserved in all the three isoforms of Orai (Fig. S4), it would be interesting to test whether D110/112A also influences pH_o_ sensitivity of Orai2/STIM1.

Taken together, we found that different amino acid residues are responsible for pH_o_ sensitivity of Orai1/STIM1 under different ionic conditions. Under physiological conditions when Ca^2+^ is the permeant cation, it appears that E106^26^ and likely D110/D112^27^ are responsible for pH_o_ sensitivity, whereas E190 contributes to pH sensitivity when Na^+^ is the charge carrier. Although changes of Ca^2+^ and Na^+^ selectivity and permeability are proposed as the underlying mechanism for Orai1/STIM1 regulation by external protons, we and others^26^,^27^ have not provided direct experimental evidence yet. Indeed, it seems that the outward currents are much larger and the reversal potential is not as positive as expected at high pH_o_ (Figs 3 and 4), suggesting that alkaline pH changes ionic selectivity and permeability. Future studies focusing on how different pH_o_ can change Ca^2+^ selectivity and permeability of WT Orai1 and its mutants, how single channel conductance of the WT and mutated channels is influenced by various pH_o_, and whether mutating E106 and E190 to different amino acid residues will alter the pH_o_ sensitivity are required to fully understand the molecular mechanisms underlying pH_o_ sensitivity of Orai1/STIM1.

### Mechanisms of intracellular pH sensitivity of Orai1/STIM1 channels

Previous studies showed that native I_CRAC_ can be blocked by internal acidic pH^16^, but the effect of basic pH was not evaluated. We demonstrate that Orai1/STIM1 channel activity is not only inhibited by acidic pH_i_, but also enhanced by alkaline pH_i_. To further understand mechanisms of internal pH regulation of Orai1/STIM1, we generated a series of mutations on the Cys, His and Glu residues located in the N- and C-termini, as well as the intracellular loop between TM2 and TM3. Among the twelve mutants, H155F displayed markedly reduced sensitivity to both acidic and alkaline pH_i_, indicating that H155 at the intracellular loop of TM2 and TM3 is responsible for sensing internal pH changes.

It is remarkable that neutralizing one residue H155 diminishes the sensitivity of Orail1/STIM1 to both acidic and alkaline pH_i_. Since the side chains of His and phenylalanine (Phe) are similar, it appears that the charge of H155 plays an essential role in sensing the internal pH changes. Although the exact mechanism is not clear yet, it is plausible that protonation of H155 under acidic pH_i_ conditions affects intra- or intermolecular interactions of the channel leading to conformational changes that favor channel closing, whereas deprotonation of H155 under alkaline pH_i_ conditions causes conformational changes that favor channel opening. In a previous study, a His residue in the N-terminus has been reported to be responsible for the alkaline pH_i_ induced activation of TRPV1^30^. TRPA1 is also activated by alkaline pH_i_^32^. However, unlike TRPV1, the mechanism by which alkaline pH_i_ activates TRPA1 is through the modulation of two N-terminal Cys residues^32^. Internal pH regulation of HCN2 channel is also mediated by a His residue located in the cytosolic S4-S5 linker^31^. Protonation of H321 causes a leftward shift of the activation curve therefore reducing HCN2 current amplitude, whereas deprotonation of H321 elicits a rightward shift of the activation curve therefore enhancing HCN2 current amplitude^31^. Several other channels are also regulated by internal pH changes. For example, TRPM2 and TRPM7 are inhibited by acidic pH_i_^48^,^56^. An intracellular residue D933 located at the TM4 and TM5 linker was found to serve as the internal pH sensor of TRPM2^48^. The residue D933 contributes to TRPM2 pH_i_ sensitivity by influencing TRPM2 channel gating as well as Ca^2+^ and ADPR sensitivity^48^. Different from the mechanism by which TRPM2 is regulated by pH_i_, intracellular protons inhibit TRPM7 channel activity by screening the negative charges of PIP_2_^56^.

How might H155 located in the loop of TM2 and TM3 sense the changes of internal pH and translate the pH sensitivity to channel gating? The TM2-TM3 loop has previously been shown to play an essential role in fast inactivation of Orai1/STIM1^57^. Mutation of the four residues in the middle of the loop abolished fast inactivation of Orai1, and addition of a 37-amino acid peptide derived from the loop blocked Orai1 currents^57^. It was proposed that the intracellular loop between TM2 and TM3 of Orai1 may function as an inactivation particle which mediates fast inactivation of Orai1/STIM1^57^. Therefore, it is conceivable that protonation and deprotonation of H155 cause conformational changes of the loop between TM2 and TM3, leading to changes of Orai1/STIM1 channel activity.

Since I_CRAC_ activation requires coupling of Orai and STIM, as well as gating of Orai1, it was previous demonstrated that acidic pH under hypoxia conditions causes uncoupling of STIM1 and Orai1 and thereby reduces current amplitude^25^. Moreover, intracellular alkalinization has been shown to inhibit Ca^2+^-ATPase (SERCA), leading to store depletion and Orai1/STIM1 activation^58^. Using high EGTA buffering condition to passively delete the store, we demonstrate that changes in internal pH directly influence pore-forming subunit Orai1 through protonation and deprotonation of H155 in the loop of TM2 and TM3, a previously unknown mechanism by which Orai1/STIM1 channel activity is regulated by pH_i_.

## Conclusions

We demonstrate that the Orai1/STIM1 channel is regulated by changes of both intracellular and extracellular pH. Acidic internal and external pH reduce Orai1/STIM1 channel activity, whereas alkaline intracellular and extracellular pH enhance Orai1/STIM1 channel activity. Whereas E106 is responsible for external pH sensitivity when Ca^2+^ is the charge carrier as previously reported, we find that the residue E190 in TM3 is the major extracellular pH sensor when Na^+^ is the permeant cation. Moreover, we demonstrate that H155 in the intracellular TM2-TM3 loop is the intracellular pH sensor of Orai1/STIM1 channels. Similar to the pH sensitivity of over-expressed Orai1/STIM1 channels, the endogenous I_CRAC_ is also regulated by changes of internal and external pH. Given the important roles of intracellular and extracellular pH in a variety of cellular functions, our results suggest that Orai1/STIM1 channels could be an essential mediator for pH induced modulation of physiological/pathological functions.

## Materials and Methods

### Molecular Biology

The constructs Orai 1–3 and STIM1 from Dr. Rao’s group^20^ were purchased from Addgene (IDs: 12199, 16369, 16370 and 19754 for Orai1, Orai2, Orai3, and STIM1, respectively). Mutations of Orai1 were generated by site-directed mutagenesis (QuickChange, Stratagene) following the manufacturer’s instruction. The predicted mutations were verified by sequencing analysis.

### Cell culture and functional expression of Orai1/STIM1 and the mutants

HEK-293 cells were grown in DMEM/F12 medium supplemented with 10% FBS, 100 U/ml penicillin and 100 mg/ml streptomycin at 37 °C in a humidity-controlled incubator with 5% CO_2_. Cells were transiently transfected with wild-type (WT) Orai1/STIM1 or its mutants by Lipofectamine 2000 (Invitrogen). We used 5 μl Lipofectamine 2000 for trasfection of the cells in a 35 mm culture dish. The GFP-containing pEGFP vector was transfected to the HEK293 cells as mock controls. Successfully transfected cells can be identified by their green fluorescence when illuminated at 480 nm. Electrophysiological recordings were conducted between 24 to 36 h after transfection. All patch-clamp experiments were performed at room temperature (20–25 °C).

RBL-2H3 cells were provided by Dr. D. Clapham (Harvard Medical School, Boston, MA). Cells were cultured in DMEM supplemented with 10% FBS and 100 U/ml penicillin and 100 mg/ml streptomycin. For electrophysiological experiments, cells were plated onto glass coverslips and used 12 h thereafter^59^.

### Electrophysiology

Whole-cell currents were recorded using an Axopatch 200B amplifier. Data were digitized at 10 or 20 kHz, and digitally filtered off-line at 1 kHz. Patch electrodes were pulled from borosilicate glass and fire-polished to a resistance of ~3 MΩ when filled with internal solutions. Series resistance (R_s_) was compensated up to 90% to reduce series resistance errors to <5 mV. Cells with R_s_ > 10 MΩ were discarded^60^.

For whole cell current recording, voltage stimuli lasting 250 ms were delivered at 1 s intervals with voltage ramps or voltage steps ranging from −120 to +100 mV. A holding potential of 0 mV was used for most experiments, unless otherwise stated. A fast perfusion system was used to exchange extracellular solutions, with a complete solution exchange achieved in about 1 to 3 s^45^. Original traces without leak subtraction were used in most figures unless otherwise stated. For the recording traces with leak subtraction (Figs 1, 2 and 4), leak currents were determined by taking an initial ramp current before Orai1/STIM1 activated^61^ or after Orai1/STIM1 currents were blocked by 10 μM La^3+^ 20,62.

The internal pipette solution for whole cell current recordings contained (in mM) 145 Cs-methanesulfonate (CsSO_3_CH_3_), 8 NaCl, 10 EGTA, and 20 HEPES, with pH adjusted to 7.2 with CsOH. MgCl_2_ (3 mM) was included in the pipette solution to block potential endogenous TRPM7 currents in the cells^63^,^64^. Acidic pH_i_ was adjusted by citrate acid with 5 MES, and alkaline pH_i_ was adjusted by CsOH.

The standard extracellular Tyrode’s solution for whole cell current recordings contained (mM): 145 NaCl, 5 KCl, 2 CaCl_2_, 1 MgCl_2_, 10 HEPES and 10 glucose; pH was adjusted to 7.4 with NaOH. Extracellular solution with acidic pH was prepared by adjusting pH with HCl and 5 mM MES; and extracellular solution with basic pH was adjusted by NMDG. Divalent-free solution (DVF) contained (mM) 145 Na-SO_3_CH_3_, 20 HEPES, 5 EGTA, 2 EDTA and 10 glucose, with estimated free [Ca^2+^]<1 nM and free [Mg^2+^] ≈10 nM at pH 7.4. The acidic pH was adjusted with citric acid and 5 mM Mes; and basic pH was adjusted using NMDG. MaxChelator was used to calculate free Ca^2+^ and free Mg^2+^ concentrations (http://www.stanford.edu/~cpatton/webmaxcS.htm). The solutions containing 2, 20, and 120 mM Ca^2+^ concentrations were prepared by reducing Na^+^ concentrations in Tyrode’s solution to keep the same osmolarity. The acidic pH was adjusted with citric acid and 5 mM Mes; and basic pH was adjusted using NMDG as previously reported^62^. The amount of NMDG used for adjusting pH in different solutions did not influence Orai1/STIM1 current amplitude. For example, the average current amplitude in pH_o_ 9.0 DVF solution adjusted with NMDG was 185.7 ± 20.3 pA/pF, and the current amplitude of Orai1/STIM1 recorded in pH_o_ 9.0 DVF solution adjusted with NaOH was 187.4 ± 19.6 pA/pF (n = 4), and there was no statistical difference (p > 0.05). All the chemicals used in electrophysiological experiments were from Sigma-Aldrich. Solutions were titrated to their nominal pH at room temperature (20–25 °C).

### Data Analysis

Pooled data are presented as mean ± SEM. Dose–response curves were fitted by an equation of the form: *E *= *E*_*max*_{1/[1+(EC_50_/*C*)^*n*^]}, where *E* is the effect at concentration *C*, *E*_max_ is maximal effect, EC_50_ is the concentration for half-maximal effect and *n* is the Hill coefficient^65^. EC_50_ is replaced by IC_50_ if the effect is an inhibitory effect. Statistical comparisons were made using analysis of variance (ANOVA) and two-tailed *t-*test with Bonferroni correction. P < 0.05 indicated statistical significance.

## Additional Information

**How to cite this article**: Tsujikawa, H. *et al.* Identification of key amino acid residues responsible for internal and external pH sensitivity of Orai1/stim1 channels. *Sci. Rep.* **5**, 16747; doi: 10.1038/srep16747 (2015).

## Supplementary Material

Supplementary Data
