# Supplementary material for: Identification of key amino acid residues responsible for internal and external pH sensitivity of Orai1/STIM1 channels
Source: Sci Rep. 2015 Nov 18;5:16747. doi: 10.1038/srep16747 (PMC4649748; doi:10.1038/srep16747)
Supplement: Supplementary Data [file srep16747-s1.doc]

**Identification of key amino acid residues responsible for internal and external pH sensitivity of Orai1/STIM1 channels**

Hiroto Tsujikawa^1,2,4^, Albert S Yu^1,4^, Jia Xie^1,3,4^, Zhichao Yue^1,4^, Wenzhong Yang^1^, Yanlin He^1^, Lixia Yue^1*^

^1^Calhoun Cardiology Center, Department of Cell Biology, University of Connecticut Health Center, Farmington, CT, USA;

^2^ Current address: Faculty of Health Sciences and Nursing, Juntendo University, 3-7-33 Omiyacho, Mishima, Shizuoka, Japan, 411-8787

^3^ Current address: The Scripps Research Institute, 10550 N. Torrey Pines Rd. MB-214, La Jolla CA, 92037

^4^ These authors contributed equally to this work

^*^To whom correspondence should be addressed: Dr. Lixia Yue, University of Connecticut Health Center, 263 Farmington Ave., Farmington, Connecticut, CT 06030; USA. Phone#: 860-679-3869; Fax#: 860-679-1426; e-mail: [lyue@uchc.edu](mailto:lyue@uchc.edu)

SUPPLEMENTARY DATA

**Supplementary Figure 1**. Orai1/STIM1 was not activated by external alkaline pH. Time-dependent changes of inward current measured at -100 mV under different conditions. In the first 6 min, current was not induced by external perfusion with tyrode solution at pH_o_ 7.2 or 8.2 under perforated patch configuration. After whole-cell configuration, Orai1/STIM1 was activated when the pipette solution dialyzed into the cell to deplete the store. NMDG solution was used to test leak current level. Similar results were obtained in another three cells (n=4).

**Supplementary Figure 2.** Effects of acidic and basic pH on non-transfected cells. Currents were elicited by the ramp protocol. There was no noticeable current induced by pH_o_ 5.5 or pH_o_ 8.2 (n=4).


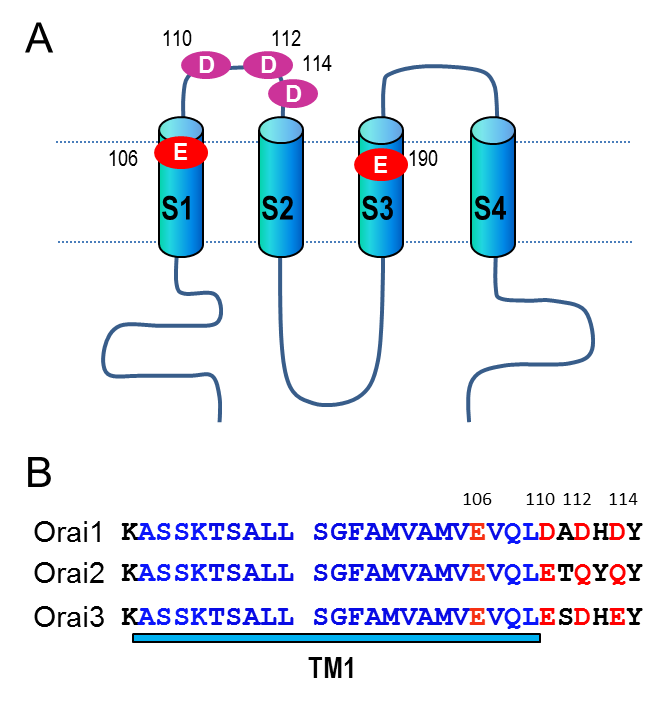


# Supplementary Figure 3. Mutations for pH_o_ sensitivity. A, Schematic diagram of the pore-forming subunit Orai1. B, alignment of the first transmembrane domain (TM1) and the extracellular TM1-TM2 loop of Orai1, Orai2, and Orai3. The residues highlighted in red are the mutated residues.

#

# Supplementary Figure 4. Original recordings at pH_i_ 5.5, 7.4 and 9.0 of the mutants H69, C143S, E149Q, H169N, H169/171F, E173Q, H256F, H264F, and E272/275Q.

#

#

# Supplementary Figure 5. Average current amplitude of WT and mutants E106D, E190D, D110N, E190Q and D112/114N measured at -100 mV in Tyrode solution containing 20 mM Ca^2+^ at pH_o_ 7.4, 5.5 and 9.0. (Mean±SEM, n=6~16; *: p<0.05; **p<0.01).
